# Uterine Displacements

**Published:** 1884-05

**Authors:** 


### BOOK NOTICES.

Uterine Displacements. By S. J. Donaldson, M. D., New York. Pp. 83. Otis Clapp & Son, Boston, Mass.

This little work gives one the best idea of the relative position of the pelvic viscera we have seen. His criticisms of the diagrams contained in "Gray" and other noted works are well timed, and had this book no other merit this would suffice to recommend it to every student and physician. It abounds in practical suggestions and justly condemns the use of the pessary. Therapeutics are omitted, as he aptly states, because "it is the 'totality of the symptoms' of each individual case, duly pondered upon, that alone can guide us in the selection of the appropriate drug remedy." F. P.

